# Multiplexed single-cell lineage tracing of mitotic kinesin inhibitor resistance in glioblastoma

**DOI:** 10.1016/j.celrep.2024.114139

**Published:** 2024-04-21

**Authors:** Yim Ling Cheng, Matei A. Banu, Wenting Zhao, Steven S. Rosenfeld, Peter Canoll, Peter A. Sims

**Affiliations:** 1 Department of Systems Biology, Columbia University Irving Medical Center, New York, NY 10032, USA; 2 Department of Neurological Surgery, Columbia University Irving Medical Center, New York, NY 10032, USA; 3 Department of Cancer Biology, Mayo Clinic, Jacksonville, FL 32224, USA; 4 Department of Pathology and Cell Biology, Columbia University Irving Medical Center, New York, NY 10032, USA; 5 Department of Biochemistry and Molecular Biophysics, Columbia University Irving Medical Center, New York, NY 10032, USA; 6 Lead contact

## Abstract

Glioblastoma (GBM) is a deadly brain tumor, and the kinesin motor KIF11 is an attractive therapeutic target with roles in proliferation and invasion. Resistance to KIF11 inhibitors, which has mainly been studied in animal models, presents significant challenges. We use lineage-tracing barcodes and single-cell RNA sequencing to analyze resistance in patient-derived GBM neurospheres treated with ispinesib, a potent KIF11 inhibitor. Similar to GBM progression in patients, untreated cells lose their neural lineage identity and become mesenchymal, which is associated with poor prognosis. Conversely, cells subjected to long-term ispinesib treatment exhibit a proneural phenotype. We generate patient-derived xenografts and show that ispinesib-resistant cells form less aggressive tumors *in vivo*, even in the absence of drug. Moreover, treatment of human *ex vivo* GBM slices with ispinesib demonstrates phenotypic alignment with *in vitro* responses, underscoring the clinical relevance of our findings. Finally, using retrospective lineage tracing, we identify drugs that are synergistic with ispinesib.

## In brief

Cheng et al. show that the kinesin motor KIF11 inhibitor ispinesib prevents mesenchymal drift of glioblastoma cells *in vivo*, leading to less aggressive tumors and improved survival. Through single-cell analysis and retrospective lineage tracing, they identify drug targets, offering a strategy to find potential drug combinations to combat resistance.

## Graphical Abstract

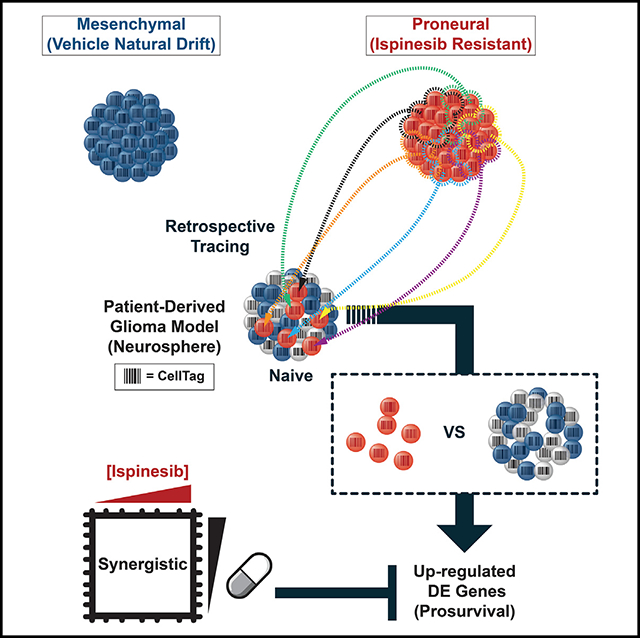

## INTRODUCTION

Failure of effective cancer treatment is due, in part, to the dynamic and evolving nature of tumors. Glioblastoma (GBM) is an incurable malignancy with a 6.9% 5-year survival rate^1^; it is a highly heterogeneous and plastic tumor that is able to traverse multiple cell states in response to extrinsic and intrinsic cues.^2–4^ Molecular profiling of GBM with single-cell RNA sequencing (scRNA-seq) and other methods has identified co-occurring cellular states that were observed across patients along with transitions between them.^2,5,6^ These states are likely differentially sensitive to treatment and can result in therapy-resistant populations that give rise to aggressive recurrent tumors.

Drug resistance can arise from both genetic selection and preexisting or adaptive transcriptional states, which lead to an advantageous phenotype.^7–12^ Only limited genetic alterations are associated with recurrence in GBM, potentially indicating a larger role of non-genetic mechanisms in GBM.^13–15^ Devising effective therapies requires tools for characterizing and identifying vulnerabilities in drug-resistant states.

Inhibitors of the kinesin-5 (also known as Eg5 and KIF11), a mitotic kinesin, are promising candidates for GBM therapy but can be rendered ineffective due to various mechanisms of resistance.^16^ Kinesin-5 is a microtubule-associated motor protein that organizes the bipolar mitotic spindle and facilitates cell migration.^17,18^ Its involvement in two hallmarks of cancer, proliferation and invasion, makes it a potential therapeutic target. GBM is deadly not only because GBM cells proliferate but also because they invade the brain microenvironment. For inhibitors to be suitable for systemic GBM therapy, they also need to exhibit low neurotoxicity and high blood-brain barrier penetration. Ispinesib is one such potent inhibitor and has been shown to prolong the survival of murine GBM models.^19,20^ Resistance to this class of inhibitors can occur through point mutations that block drug binding,^21^ upregulation of an alternative kinesin,^22^ upregulation of drug efflux transporters,^20^ upregulation of EGF (epidermal growth factor) to promote cell cycle progression,^23^ and activation of STAT3 (signal transducer and activator of transcription 3) to inhibit apoptosis.^24^

To study the dynamics of GBM resistance and identify potential drug combination targets, we transduced PDGFRA (platelet-derived growth factor receptor alpha)-amplified, patient-derived glioma neurospheres (TS543^25^) with a barcoded lineage-tracing library (CellTag^26^), and treated the neurospheres with ispinesib. These genetically modified, patient-derived neurospheres recapitulate key aspects of GBM heterogeneity and allow for surveillance of resistant phenotypes on multiple timescales. Lineage-tracing barcodes allow us to selectively analyze clones that are destined for resistance in the drug-naive setting. We analyzed the phenotypes of the glioma cells during long-term ispinesib treatment with scRNA-seq; assessed the stability and survival impact of drug-resistant phenotypes in the absence of drug and inorthotopic xenografts, complemented by slice culture experiments that demonstrated that ispinesib-treated GBM patient slice cultures phenotypically align with the TS543 ispinesib-resistant phenotype; and identified molecular markers of prospective resistant clones in the drug-naive setting to nominate effective drug combinations.

## RESULTS

### The kinesin-5 inhibitor ispinesib prevents mesenchymal transformation, resulting in a proneural resistant population

**Figure 1.**
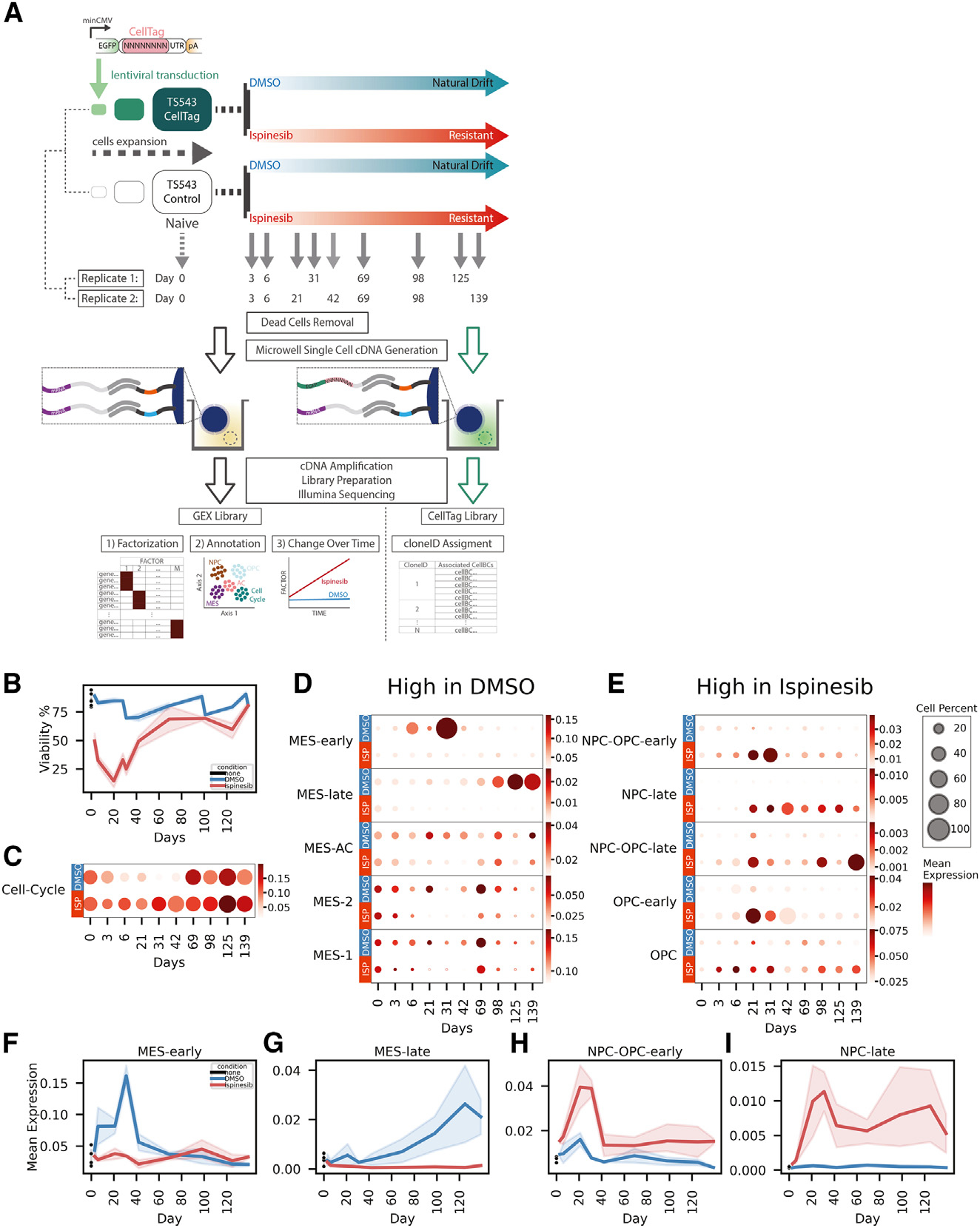
Ispinesib prevents mesenchymal transformation, and the resistant population harbors a proneural phenotype (A) Experimental schematic of single-cell lineage tracing and ispinesib-resistance time course in TS543 glioma neurospheres. (B) Viability of TS543 during treatment. Data are represented as mean ± 95% confidence interval (CI) based on four biological replicates. (C–E) Dot plots of cell cycle factor (C), MES-/AC-associated factors (D), and NPC-/OPC-associated factors (E) with size indicating the percentage of cells with high cell scores for the factor and color gradient indicating the mean log-normalized gene expression of top-ranked genes of the factor. (F–I) Line plots of mean log-normalized gene expression of top-ranked genes in MES-early factor (F), MES-late factor (G), NPC-OPC-early factor (H), and NPC-late factor (I). Data are represented as mean ± 95% CI based on four biological replicates. See also Figure S1–S3 and Tables S1 and S2.

We first sought to compare the phenotypes of glioma cells after long-term treatment with ispinesib and DMSO (vehicle). We performed long-term treatment experiments (Figure 1A) as follows: (1) small replicate populations of TS543 were seeded and either transduced with CellTag (celltag1 and celltag2),^26^ a lentivirus barcode library, or not (control1 and control2); (2) the cells were expanded prior to treatment; (3) scRNA-seq was performed on the drug-naive cells; (4) after drug treatment started, scRNA-seq was initially performed at 3-day intervals and later at 2- and 4-week intervals; and (5) viability of cells was monitored (Figure 1B) during this period to monitor drug resistance. We also performed whole-exome sequencing on genomic DNA samples of ispinesib-resistant and DMSO-natural-drift cells (STAR Methods) but did not detect single-nucleotide variants (Table S2) in genes associated with known resistance mechanisms for this class of inhibitors.^20–24^

To identify cellular states and temporal patterns in the resulting dataset, we first pooled scRNA-seq data from all four replicates (control1, control2, celltag1, celltag2) and all time points to construct a factor model using single-cell hierarchical Poisson factorization (scHPF),^27^ a probabilistic algorithm for identifying co-expression signatures from scRNA-seq. scHPF computed scores that rank the association of each gene (Figure S1A; Table S1) and cell with each identified co-expression signature or factor. We noticed that the highly ranked genes in many of the factors correspond to gene signatures reported in previous single-cell studies of GBM.^2^ Thus, we systematically identified factors with high gene scores for the cellular subtype gene sets from Neftel et al.^2^ (Figure S1B). These gene sets were derived from the integration of scRNA-seq from 28 GBM and 401 bulk RNA-seq profiles from The Cancer Genome Atlas and correspond to cell cycle (G1/S, G2/M), hypoxic and non-hypoxic mesenchymal (MES)-like (MES-1 and MES-2, respectively), astrocytic (AC)-like, oligodendrocyte precursor (OPC)-like, and neural progenitor (NPC)-like (NPC1 and NPC2).^2^ For factors exhibiting a clear temporal pattern, we appended the term “early” to the factor annotation if the factor primarily has high expression before day 42 or “late” if it mainly has high expression after day 42. In total, we annotated 11 factors: cell-cycle-1, cell-cycle-2, NPC-late, NPC-OPC-late, NPC-OPC-early, OPC-early, OPC, MES-early, MES-1, MES-2, MES-late, and MES-AC (Figure S1B; Figures 1C–1E); the top-ranked genes of cell-cycle-1 and cell-cycle-2 were combined into a single cell cycle factor.

Across our time course, we observed a clear enrichment of MES-associated factors in the DMSO samples (Figure 1D) and NPC-/OPC-associated (proneural) factors in the ispinesib-treated samples (Figure 1E). Specifically, we observed emergence of the MES-early cell state followed by a gradual increase in the MES-late cell state in the DMSO time course (Figures 1D, 1F, and 1G). In contrast, we observed an early emergence in NPC-OPC-early and OPC-early cell states followed by an increase of NPC-late and NPC-OPC-late cell states for ispinesib-treated samples (Figures 1E, 1H, and 1I). These results were reproducible among the four replicates (Figures S2A and S2B). To visualize the similarity of the four replicates, we also embedded the DMSO and ispinesib samples separately into a low-dimensional space with PHATE.^28^ PHATE embeddings for the DMSO and ispinesib datasets were also reproducible across the four replicates (Figures S3A–S3D). The dominant temporal patterns are drifts of the DMSO- and ispinesib-treated cells toward MES and NPC states, respectively.

The drift toward a MES phenotype is a common phenomenon in solid tumors including GBM,^29–33^ where it is associated with recurrence, drug resistance, and poor prognosis.^34^ While it is unsurprising to observe MES drift in the DMSO time course as described above, this does not occur in the ispinesib-treated populations. Thus, although glioma cells develop resistance to ispinesib, the resistant cells resemble the proneural glioma phenotype, which is associated with improved survival.^35,36^

### The proneural phenotype of ispinesib-resistant clones is preserved in drug-free xenograft and associated with better survival

**Figure 2.**
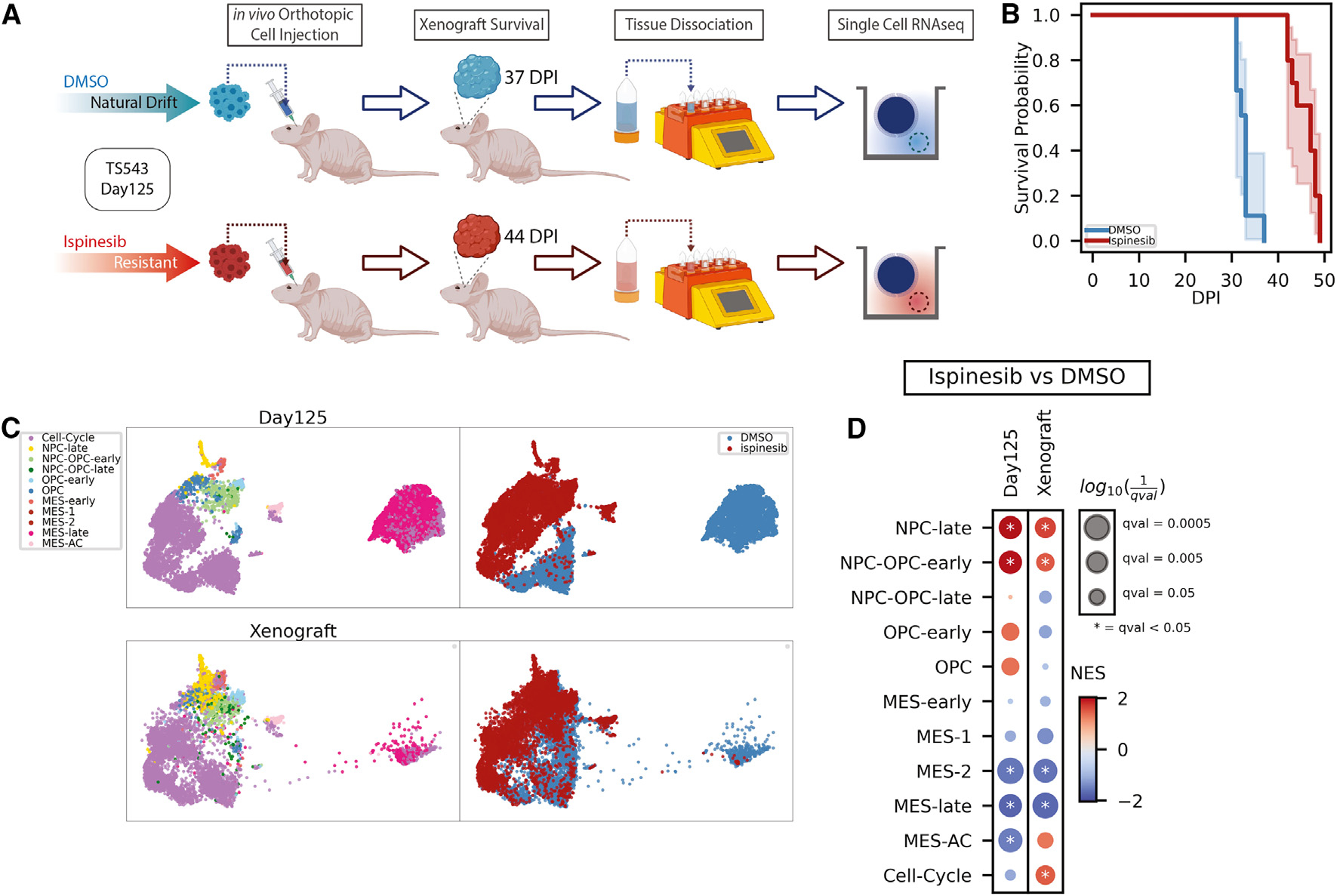
Proneural phenotype of ispinesib-resistant clones is preserved in drug-free xenograft and provides survival advantage (A) Experimental schematic of scRNA-seq of xenografts derived from DMSO-treated and ispinesib-resistant clones of last *in vitro* time point (day 125 control1 samples). (B) Survival probabilities of ispinesib-resistant and DMSO-natural-drift clone-derived xenograft models are presented with Kaplan-Meier curves ± 95% CI based on ten mice per cohort (p = 8 × 10^−6^, log-rank test). (C) UMAP embeddings of last *in vitro* time point scHPF factors and xenograft dataset projection onto the scHPF model of the *in vitro* time course color coded by cell states and treatments. (D) Normalized enrichment score (NES) from gene sets derived from top-ranked genes in single-cell hierarchical Poisson factorization (scHPF) and pre-rankeddifferential expression analysis results between ispinesib and DMSO samples. Significance is reported as false discovery rate (FDR) q-values from the GSEA. See also Figures S4 and S5.

Our scRNA-seq time course revealed that ispinesib-resistant cells become increasingly proneural over time, whereas DMSO cells drift toward a MES phenotype associated with recurrence and poor survival.^37,38^ Thus, we hypothesized that, unlike drug-resistant cells in many other settings, ispinesib-resistant cells might form less aggressive tumors than the untreated population. To test this hypothesis, we generated xenograft models by orthotopic transplantation of ispinesib-resistant and DMSO-natural-drift cells from the last *in vitro* time point (day 125 control1 samples) (Figure 2A). Importantly, these xenografts were formed in the absence of drug. As expected, xenografted mice derived from ispinesib-resistant cells have a significant survival advantage (median survival: 33 and 47 days for xenografts from DMSO- and ispinesib-treated cells, respectively; p = 8 × 10^−6^, log-rank test; Figure 2B), with no mice injected with ispinesib-resistant cells dying before any of the mice injected with DMSO-natural-drift cells.

Given that these xenografts formed over the course of several weeks in the brain microenvironment and in the absence of drug, we next asked whether the phenotypic differences between the DMSO-natural-drift and ispinesib-resistant cells were preserved in end-stage tumors. We performed scRNA-seq on end-stage tumors from each population. To compare the phenotypes between the end-stage tumors of xenografts and the last *in vitro* time point from which these xenografts were derived, we projected the scRNA-seq data from xenografts into the scHPF model of the original *in vitro* time course and visualized the xenograft projection along with the last *in vitro* time point with uniform manifold approximation and projection (UMAP) (Figure 2C). The ispinesib-resistant and DMSO xenograft profiles project onto the *in vitro* ispinesib-resistant and DMSO-natural-drift populations, respectively, suggesting that key aspects of the ispinesib-resistant and DMSO-natural-drift phenotypes are preserved *in vivo* in the absence of treatment (Figure 2C).

To confirm that the MES phenotype of DMSO-natural-drift cells and the proneural phenotype of ispinesib-resistant cells were maintained in the xenograft, we compared the two populations using differential expression analysis. We then used gene set enrichment analysis (GSEA) to identify scHPF signatures that are statistically enriched among the differentially expressed genes (Figure 2D). NPC-OPC-early and NPC-late signatures of ispinesib-resistant cells and MES-2 and MES-late signatures of DMSO-natural-drift cells are maintained in the xenograft (Figure 2D). Thus, despite growing in the absence of ispinesib for more than a month, ispinesib-resistant cells maintain their MES-depleted, proneural-enriched phenotype, consistent with the observed enhanced survival.

To identify the molecular alterations accompanying the transition from *in vitro* to *in vivo*, we performed differential expression analysis and GSEA to compare an ispinesib-resistant-derived xenograft against its *in vitro* counterpart. As expected, we observed significant depletion of gene sets related to mitochondrial complexes, oxidative phosphorylation, and lipid metabolism *in vivo* for both ispinesib-resistant glioma cells and DMSO-natural-drift glioma cells, consistent with a general adaptation to metabolic demands and reduced oxygen availability *in vivo* (Figure S4). Notably, enrichment of gene sets related to cellular stress, PDGF signaling, and protein metabolism that underscore the expected influence of the disparity in growth factors and the metabolic adjustments required for growth and survival *in vivo* are only detected in DMSO-natural-drift glioma cells (Figure S4). The DMSO-natural-drift glioma cells exhibit more extensive alterations in gene expression profiles in comparison to their ispinesib-resistant counterparts. This observation underscores the likelihood that DMSO-natural-drift cells, having diverged from their neural lineage, face greater challenges in accommodating brain milieu.

Although the xenograft models are immunodeficient, there is still representation of myeloid cells, which are the most abundant component of the immune microenvironment in GBM. To understand the two xenografts’ microenvironments, we conducted scHPF analysis on the myeloid cells, which revealed enrichment of a hypoxia myeloid factor in the ispinesib-resistant-derived xenograft’s myeloid cells and an angiogenic inflammatory myeloid factor in the DMSO-nature-drift-derived xenograft’s myeloid cells. UMAP embedding of scHPF factors shows high cell scores of a hypoxia myeloid factor on the ispinesib-resistant-derived xenograft’s myeloid cells and high cell scores of an angiogenic inflammatory myeloid factor on the DMSO-natural-drift-derived xenograft’s myeloid cells (Figure S5B). GSEA on the differentially expressed genes between the ispinesib-resistant-derived xenograft’s myeloid cells and the DMSO-natural-drift-derived xenograft’s myeloid cells confirms the significant enrichment of a hypoxia myeloid factor and the depletion of an angiogenic inflammatory myeloid factor (Figure S5C). The hypoxia myeloid factor is linked to hypoxia, glycolysis, cholesterol homeostasis, and bone marrow-derived macrophages^39^ (Figures S5D and S5E), signifying an adaptation of a myeloid population to the hypoxic microenvironment. Conversely, the angiogenic inflammatory myeloid factor is associated with angiogenesis, inflammatory response, and cell communication (Figures S5D and S5E).

In conclusion, the proneural-enriched and MES-depleted phenotype of ispinesib-resistant neurospheres is manifested in xenograft models in the absence of drug, underpinning the survival advantage in an *in vivo* context. Differential expression analysis between *in vivo* vs. *in vitro* further revealed that DMSO-natural-drift cells, with the MES phenotype, undergo more pronounced gene expression changes, suggesting a greater challenge in adapting to the brain microenvironment compared to the proneural ispinesib-resistant cells.

### Ispinesib-treated GBM patient slice cultures share phenotypic features with TS543 ispinesib-resistant phenotype

**Figure 3.**
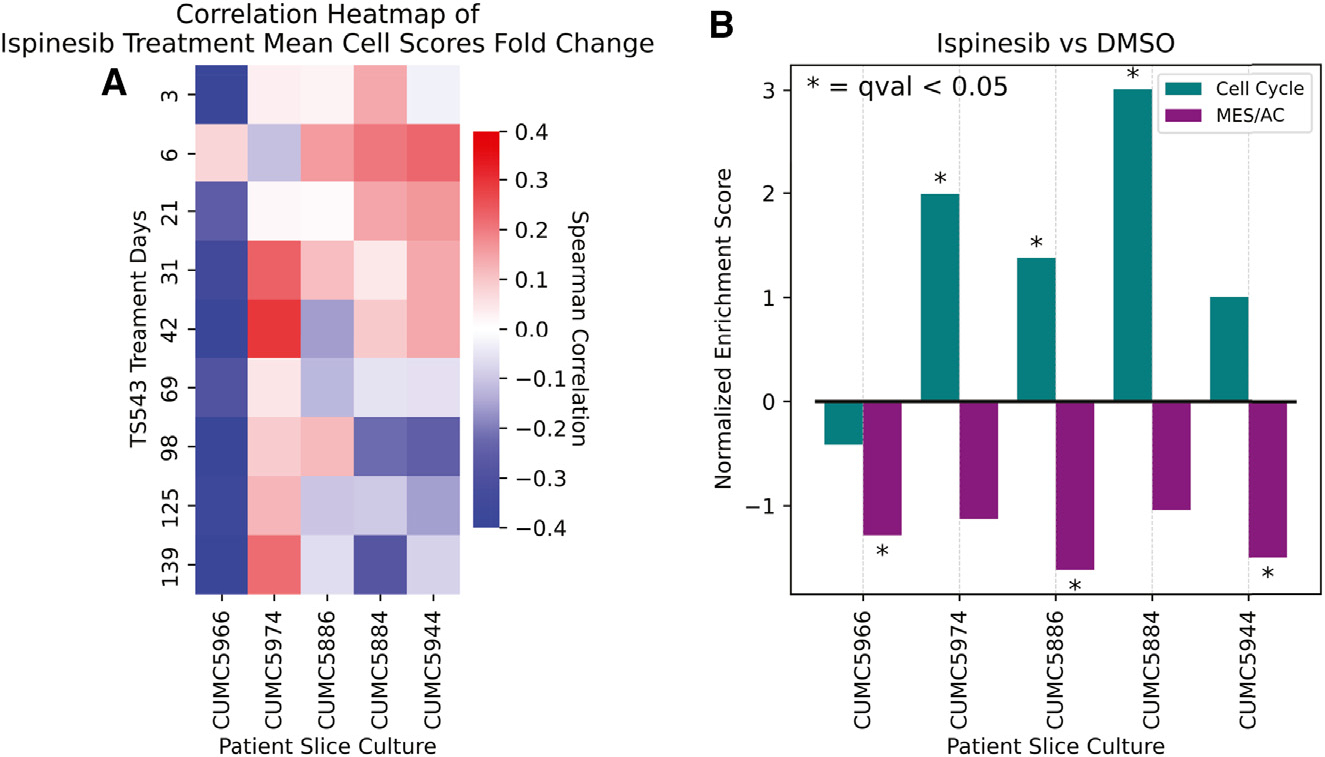
Phenotypic alignment and differential gene expression signature of ispinesib-treated GBM slice cultures reflects TS543 ispinesib-resistant phenotype (A) Heatmap of Spearman correlation between ispinesib-treatment-induced fold changes in average scHPF cell scores across five patient-derived GBM slice cultures with the time points of the longitudinal TS543 ispinesib treatment study. Fold change is the log ratio of the mean cell scores of cells treated with ispinesib to those treated with vehicle. (B) Pre-ranked GSEA results for differentially expressed genes comparing ispinesib-treated to DMSO-treated patient GBM slice cultures. Enrichment scores are shown for gene sets derived from the TS543 neurosphere model’s scHPF factors related to MES/AC and the cell cycle. Significance is reported as FDR q-values from the GSEA. See also Table S3.

In a more clinically relevant model that better mirrors the complexity of GBM in patients, slice cultures from human GBM surgical specimens were treated with ispinesib for 18 h, and scRNA-seq was performed.^40^ To place these acute treatment experiments in the context of our long-term time course in TS543 neurospheres, we projected the patient slice culture data into the scHPF model of the time course after extracting the profiles of transformed GBM cells. Figure 3A shows the correlation between each slice culture projection and time point in the TS543 ispinesib time course. Slice cultures from four of the five patients were positively correlated with early treatment stages of TS543 (Figure 3A). Interestingly, one of the five patients exhibited a negative correlation, and this same patient also exhibited resistance to etoposide, a different chemotherapy drug that targets topoisomerase II and eliminates cycling GBM cells, in our previously reported study.^40^ GSEA further elucidates this distinction (Figure 3B), showing depletion of a MES/AC signature and enrichment of cell cycle signatures in four patients’ slice cultures, consistent with the TS543 ispinesib-resistant phenotype, except for the outlier patient’s slice culture. These results highlight the relevance of the expression patterns uncovered by our time course in human patient specimens and the heterogeneity of cell-type-specific drug responses in GBM.

### Ispinesib-resistant clones are phenotypically diverse in the drug-naive setting

**Figure 4.**
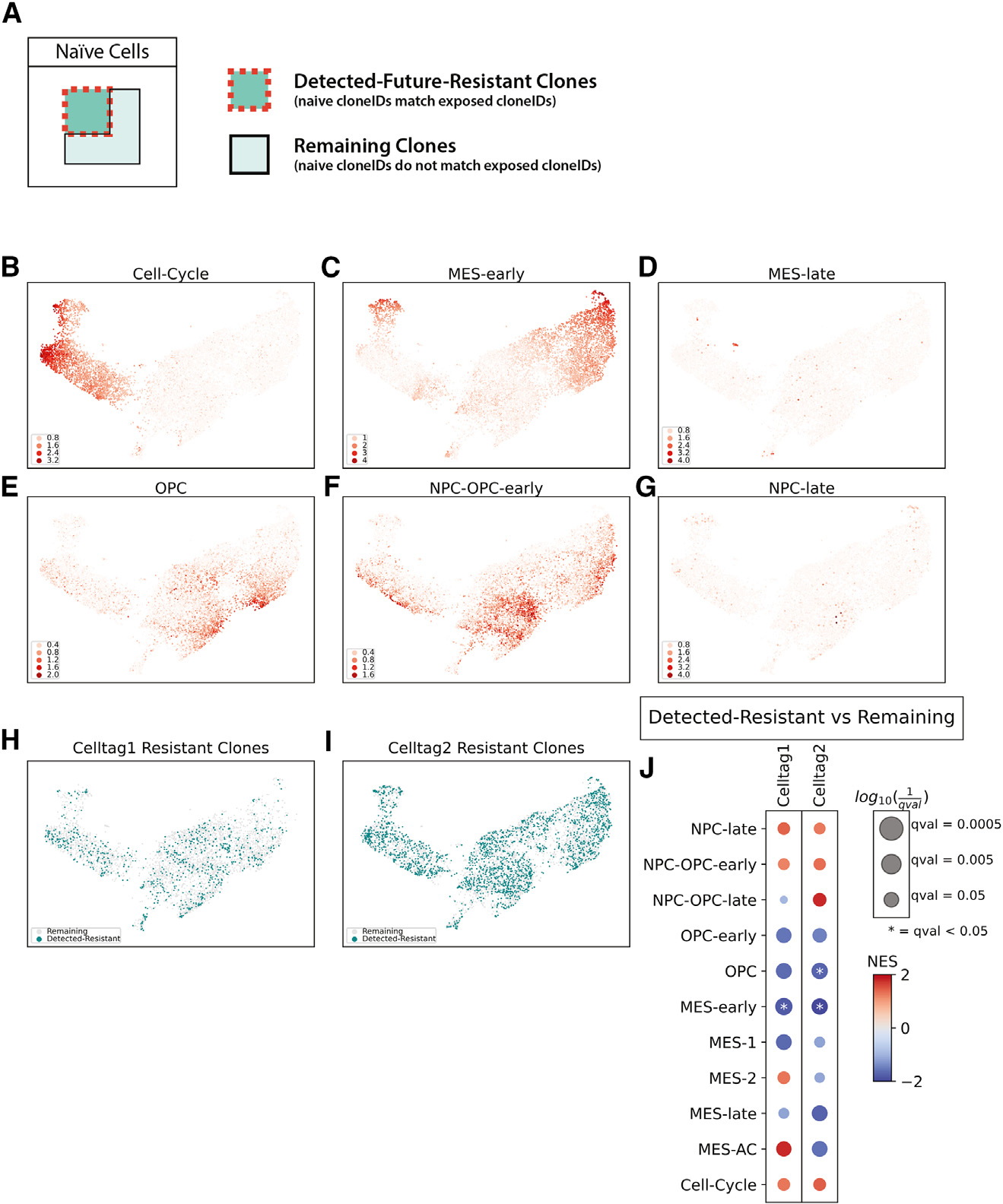
Ispinesib-resistant clones are phenotypically diverse in the naive setting (A) Definitions of detected-future-resistant clones and remaining clones. CloneIDs are the assigned IDs of the multiplexed CellTag barcodes in clones. (B–G) UMAP embedding of scHPF factors of naive cells colored by the cell scores of cell-cycle factor (B), MES-early factor (C), MES-late factor (D), OPC factor (E), NPC-OPC-early factor (F), and NPC-late factor (G). (H and I) Detected-future-resistant clones in celltag1 and celltag2 naive cells on the UMAP embedding. (J) NES of gene sets created from scHPF top-ranked genes and pre-ranked gene lists generated from differential expression analysis results between detected-future-resistant clones and remaining clones. Significance is reported as FDR q-values from the GSEA.

The CellTag barcodes allow us to retrospectively analyze the phenotype of the resistant clones in the drug-naive setting and determine whether they have unique properties relative to the remaining cells. To characterize the cell states of clones that would become resistant, we partitioned the naive samples into detected-future-resistant clones and remaining clones by matching the cloneIDs (assigned IDs of the multiplexed CellTag barcodes in clones) in the naive samples with the cloneIDs in the ispinesib-exposed samples (Figure 4A). We used the term “remaining” instead of the term “sensitive” to name the naive clones that did not have matched cloneIDs in ispinesib-exposed samples because of the possibility that not all ispinesib-exposed resistant clones were sequenced due to limited cell sampling. We embedded the scHPF factors of naive samples in two dimensions with UMAP to visualize any bias in the cell states occupied by the detected-future-resistant clones. In the UMAP embedding, we have the proliferating cells on the left side (Figure 4B). The quiescent population on the right side includes cells enriched in the OPC- and NPC-like signatures (Figures 4E–4G), with more MES (MES-early) cells on the far right (Figures 4C and 4D). The detected-future-resistant clones are distributed throughout the UMAP, with no visually obvious bias in the distribution of cell states for the detected-future-resistant clones (Figures 4H and 4I). To analyze this more quantitatively and determine if there are any significant cell state differences between detected-future-resistant clones and remaining clones, we used GSEA to identify scHPF factors with enrichment among the genes that were differentially expressed between the detected-future-resistant clones and remaining clones (Figure 4J). Depletion of the MES-early factor is significant for both replicates (celltag1 and celltag2) (Figure 4J). While the other factors do not exhibit significant enrichment or depletion in the resistant clones, many of them have reproducible patterns such as enrichment of NPC-late, NPC-OPC-early, and cell-cycle factors and depletion of MES-late and MES-1 factors (Figure 4J).

While the cells exhibit a strong phenotype after long-term treatment, there is no dominant phenotype for the detected-future-resistant clones in the naive setting and only subtle phenotypic differences between detected-future-resistant and remaining clones. This could be because the treatment-naive cells are highly plastic, with cells transitioning between these states on relatively short timescales, or because acquisition of ispinesib-resistance states is stochastic. Nonetheless, the significant and reproducible depletion of the MES-early state in the resistant clones is consistent with the depletion of MES expression signatures in the resistant cells observed at later time points.

### Identification of synergistic drug targets from gene expression analysis of ispinesib-resistant clones in the drug-naive setting

In GBM treatment, the standard-of-care agent temozolomide has demonstrated only modest efficacy, with a survival benefit of ~2.5 months.^41,42^ Its inability to eradicate GBM underscores the urgent need for improved therapeutic strategies.

With the limitations of temozolomide in mind, we explored the drug interaction between temozolomide and ispinesib. To systematically evaluate this interaction, we utilized the MuSyC^43^ (multi-dimensional synergy of combinations) framework, a two-dimensional extension of the traditional single-drug Hill equation that has five additional parameters that quantify drug interactions: efficacy (β), potency (α_12_, α_21_), and cooperativity (γ_12_, γ_21_). The intention of drug combination treatment is to reduce toxicity by minimizing dose, to improve survival by increasing efficacy, or both. The parameter α quantifies the change in effective dose (EC_50_) of the first drug in the presence of the second drug. For synergistic potency (α > 1 or log(α) > 0), the EC_50_ of the first drug decreases in the presence of the second drug, which corresponds to an increase of potency. α_21_ is the fold change in potency of drug1 induced by the presence of drug2, and vice versa. The parameter β quantifies the change in the maximal effect of the two drugs in combination compared to the maximal effect of the most efficacious single drug. For synergistic efficacy (β > 0), the combined effect at the maximum concentration tested for both drugs is greater than the maximum effect of either drug alone. Our MuSyC analysis revealed a notable antagonistic relationship when ispinesib was combined with temozolomide. Co-administration of temozolomide with ispinesib to TS543 resulted in a diminished therapeutic potency compared to either drug alone, as shown in the MuSyC delta heatmap and sideby-side dose-response curves (Figure S6).

**Figure 5.**
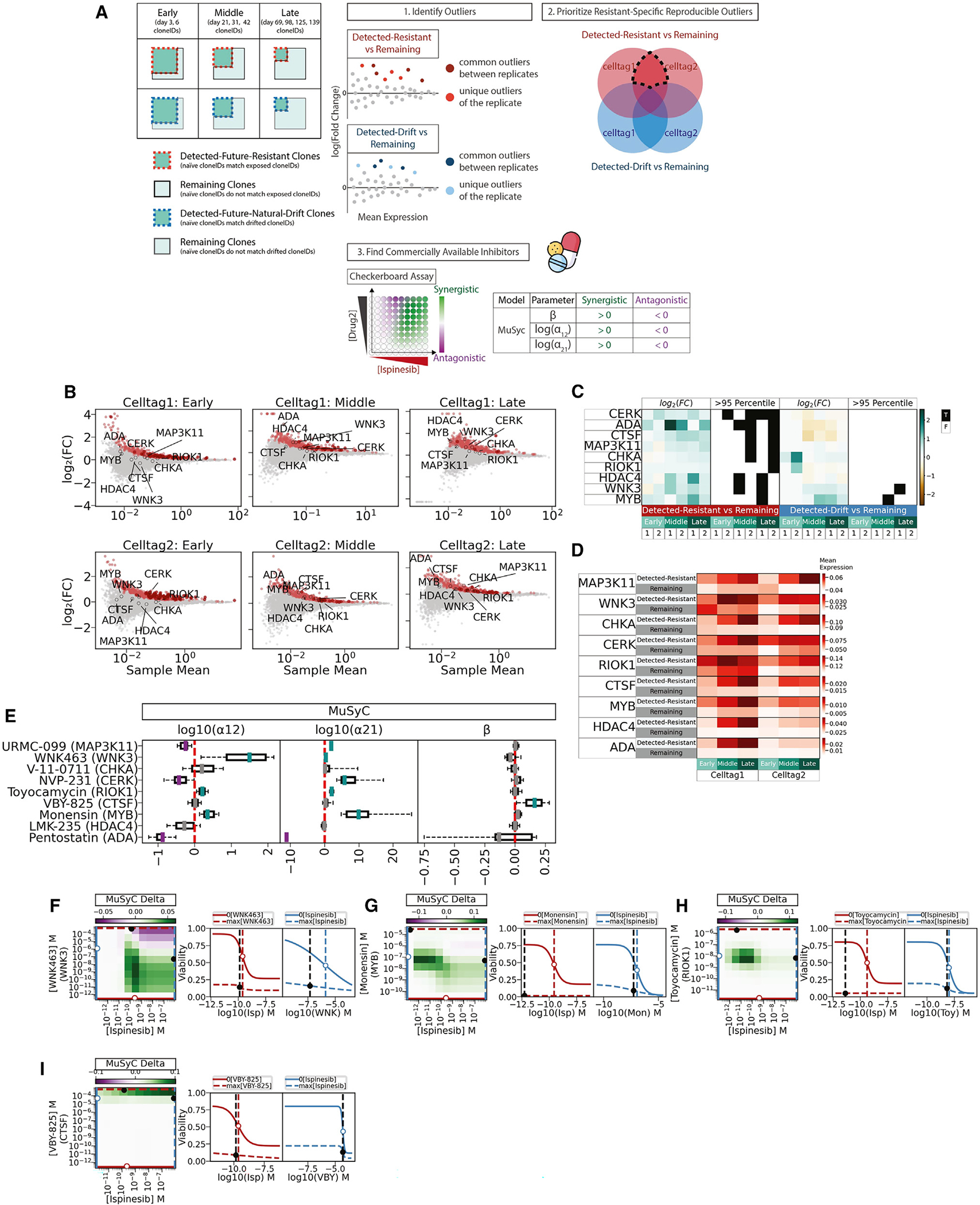
Identification of synergistic drug targets from gene expression analysis of ispinesib-resistant clones in the drug-naive setting (A) Analysis scheme for identifying potential drug combinations with ispinesib. (B) MA plots of log_2_(fold change) and sample mean from the differential expression analysis between detected-future-resistant clones and remaining clones of naive cells. Genes with fold changes that were significantly high (>95th percentile) given their sample means are colored red. (C) Heatmaps of log_2_(fold change) from the differential expression analysis between detected clones and remaining clones of naive cells. Boolean heatmaps of whether the log_2_(fold change) was significantly high (>95th percentile) given the genes’ sample means. In the x-axis labels of heatmaps, 1 is celltag1 sample, 2 is celltag2 sample, detected-resistant stands for detected-future-resistant clones, and detected-drift stands for detected-future-natural-drift clones. (D) Mean log-normalized gene expression of the druggable target genes of detected-future-resistant and remaining clones of naive cells. (E) Boxplots of synergistic parameters of MuSyC models. Parameters are presented as median values with 95% bootstrap confidence intervals based onbootstrap resampling. (F–I) Heatmaps of MuSyc delta, which is difference between the two-dimensional Hill-fitted model of the observed efficacy and the null hypothesis. Side-by-side single-drug response curve (solid curve line, with the other drug concentration at zero) and combined-drug response curve (dashed curve line, with the other drug concentration at the maximum tested). See also Figure S6.

We turned to lineage-tracing analysis via scRNA-seq to identify potential drug targets to be used in combination with ispinesib. We retrospectively lineage traced the ispinesib-resistant clones back to drug-naive cells, uncovering key genes that could serve as druggable markers for resistance. Treatment time was divided into three periods: early (6 days and earlier), middle (21–42 days), and late (69 days or later) (Figure 5A). CloneIDs of each period were used to identify clones in naive cells (Figure 5A). For each time period, we performed differential expression analysis between detected-future-resistant clones and remaining clones among drug-naive cells (Figure 5B). Differentially expressed genes were ranked by fold change and consistency between replicates (Figure 5C). Genes were removed if they were (1) also differentially expressed in clones that were selected by DMSO or (2) encoding a protein for which a commercial inhibitor was unavailable (Figure 5C). We further identified genes with increasing expression during our time course (Figure 5D). Ultimately, we identified nine gene targets with available inhibitors that we could test in combination with ispinesib. By targeting these markers, we aimed to find effective combination therapies with ispinesib. We performed a drug interaction experiment with the “checkerboard” assay and analyzed the results with MuSyc. WNK463, toyocamycin, and monensin, which are inhibitors of WNK3 (lysine-deficient protein kinase 3), RIOK1 (RIO kinase 1), and MYB (MYB proto-oncogene transcription factor), respectively, have synergistic potencies when combined with ispinesib; log(α_12_) and log(α_21_) are above zero (Figure 5E). VBY-825, an inhibitor of CTSF (cathepsin F), when combined with ispinesib, has synergistic efficacy; β is greater than zero (Figure 5E). The heatmap of MuSyc delta, which is the difference between the two-dimensional Hill-fitted model of the observed efficacy and the null hypothesis, shows regions where the combined dosage of two drugs has efficacy greater than the null hypothesis (Figures 5F–5I). The side-by-side dose-response curves show the single-drug response curve (solid curve line, with the other drug concentration at zero) and combined-drug response curve (dashed curve line, with the other drug concentration at the maximum tested). Ispinesib alone reaches maximum efficacy of 25%–20% viability (red solid curve lines), and the viability is further decreased with the addition of the second drug (blue dashed curve lines) (Figures 5F–5I). WNK3,^44–46^ RIOK1,^47–49^ MYB,^50,51^ and CTSF^52^ may protect ispinesib-resistant cells from apoptosis or help ispinesib-resistant cells progress through cell cycle. Overall, these results demonstrate how lineage-tracing analysis by scRNA-seq can be used to identify drug combinations by identifying markers of resistant clones in the drug-naive setting.

## DISCUSSION

GBM often contains cells with a MES phenotype that is associated with poor survival and drug resistance and becomes more pronounced upon recurrence. Similarly, the glioma neurosphere model used here drifts toward a MES phenotype in the absence of treatment. We found that the kinesin-5 inhibitor ispinesib effectively prevents this MES transition and that the resistant population that emerges instead harbors a proneural phenotype. Thus, we reasoned that the ispinesib-resistant cells would form less aggressive tumors than the more MES cells observed in the absence of drug. While targeted therapies often select for more aggressive phenotypes, we found that the ispinesib-resistant clones formed significantly less aggressive orthotopic xenografts, even in the absence of drug. Subsequent scRNA-seq analysis confirmed that the phenotypic differences between the ispinesib-resistant and DMSO-treated cells were largely preserved in the animal model. These findings raise the exciting possibility that not only could ispinesib serve as an effective targeted therapy in GBM but that the ispinesib-resistant clones that arise may be less aggressive than the MES cells typically found after standard treatment.

Despite the less aggressive phenotype of ispinesib-resistant cells, it remains desirable to identify drug combinations with the potential to minimize resistance. The single-cell lineage-tracing approach used here provides the ability to retrospectively analyze the phenotype of ispinesib-resistant clones in a treatment-naive population. This analysis identified genes that were enriched in drug-naive clones that would become resistant to ispinesib, some of which encoded druggable protein targets. Perhaps not surprisingly, subsequent validation experiments showed that targets associated with cell survival and apoptosis such as WNK3, RIOK1, MYB, and CTSF were synergistic with ispinesib. Previous studies of ispinesib resistance by Kenchappa et al.^24^ also concluded that glioma cells activate anti-apoptotic mechanisms to survive the prolonged G2M block produced by ispinesib, whereas normal cells apoptose under these conditions due to “mitotic catastrophe.” This phenomenon was shown to be mediated by STAT3 through its transcriptional activity and effects on mitochondrial membrane permeability and oxidative metabolism. Taken together, these studies show that multiple mediators of apoptosis could potentially be exploited by glioma cells to resist anti-mitotic drugs. Further efforts with long-term treatment and survival studies in animal models will be required to establish the pre-clinical efficacy of these interesting drug combinations. Nonetheless, the strategy employed here for discovering these drug combinations has significant advantages over conventional combinatorial screening in rapidly narrowing the scope of potential candidates targeted to drug-resistant clones.

### Limitations of the study

Our study uses a variety of patient-derived models that each have specific limitations. The TS543 neurospheres, whether tested *in vitro* or *in vivo* in xenografted, immunocompromised mice, may not fully recapitulate the complexities of ispinesib responses in patients. The *in vivo* model lacks a fully functional immune system, whereas the *in vitro* model lacks the brain microenvironment altogether. In addition, these models may not completely represent both the inter- and intra-tumoral heterogeneity observed in patients with glioma. Patient-derived slice culture, which comprises the patient’s unique tumor microenvironment and inherent genetic and cellular diversity, resolves these issues.^40^ However, we cannot perform long-term resistance studies in slice cultures and thus can only analyze acute responses to drugs. Furthermore, understanding ispinesib’s behavior and effectiveness in the brain is critical, requiring thorough pharmacokinetic and pharmacodynamic analyses and ultimately evaluation in clinical trials. Nonetheless, by performing detailed, single-cell-resolved characterization of ispinesib responses across experimental paradigms has yielded valuable results that may reflect the expected course of resistance to ispinesib or other mitotic kinesin inhibitors in GBM.

## STAR★METHODS

Detailed methods are provided in the online version of this paper and include the following:

### RESOURCE AVAILABILITY

#### Lead contact

Further information and requests for resources and reagents should be directed to and will be fulfilled by the lead contact, Peter A. Sims (pas2182@columbia.edu).

#### Materials availability

This study did not generate new unique reagents.

#### Data and code availability

- Single-cell RNA-seq data have been deposited at GEO and are publicly available as of the date of publication. Accession numbers are listed in the key resources table.
- This paper does not report original code.
- Any additional information required to reanalyze the data reported in this work paper is available from the lead contact upon request.

### EXPERIMENTAL MODEL AND STUDY PARTICIPANT DETAILS

#### Animals

All procedures were reviewed and approved by the Columbia University Institutional Animal Care and Use committee (IACUC). Nude CrTac:NCr-Foxn1^nu^ female mice (Taconic Biosciences) were used as background for *in vivo* orthotopic cell injection experiments. Mice were housed in pathogen-free facilities at Columbia University Irving Medical Center. Mice were ordered and housed under standard conditions after arrival.

#### Cell lines

PDGFRA-amplified, patient-derived glioblastoma neurospheres, TS543,^25^ were cultured with NeuroCult NS-A Proliferation Kit Human from STEMCELL Technologies. HEK293T were cultured with DMEM containing 10% FBS and 2 mM L-glutamine.

#### Human glioma patient *ex vivo* tissue slices

This work was approved by the Columbia University Irving Medical Center Institutional Review Board before commencing the study. All tumor specimens were procured from surgeries at Columbia University Irving Medical Center. Patient diagnosis information can be found in Table S3. Tumor specimens were collected immediately after surgical removal and kept in ice-cold artificial cerebrospinal fluid (ACSF) solution containing 210 mM sucrose, 10 mM glucose, 2.5 mM KCl, 1.25 mM NaH_2_PO_4,_ 0.5 mM CaCl_2_, 7 mM MgCl_2_, and 26 mM NaHCO_3_ for transportation. Patient *ex vivo* tissue slices were prepared as follows.^40^ The collected tumor specimens were placed in a drop of ice-cold ACSF and sliced using a tissue chopper (McIlwain) at a thickness of 500 μm under sterile conditions. The slices were immediately transferred to the ice-cold ACSF solution in 6-well plates using a sterile plastic Pasteur pipette half filled with ice-cold ACSF solution followed by a 15-min recovery in ACSF to reach room temperature. Intact and well-shaped slices (approximately 5–10-mm diameter) were then placed on top of a porous membrane insert (0.4 μm, Millipore). Then the membrane inserts were placed into 6-well plates containing 1.5 mL maintenance medium consisting of F12/DMEM (Gibco) supplemented with *N*-2 Supplement (Gibco) and 1% antibiotic-antimycotic (ThermoFisher). To ensure proper diffusion into the slice, culture medium was placed under the membrane insert without bubbles. A drop of 10 μL of culture medium was added directly on top of each slice to prevent the slice surface from drying. The slices were rested for 6 h with the maintenance medium in a humidified incubator at 37°C and 5% CO_2_ before drug treatment.

### METHOD DETAILS

#### CellTag barcode lentivirus packaging

CellTag barcode lentivirus was packaged according to the online protocol on protocols.io^64^ as follows. Lentiviral pSMAL-CellTag-V1 pooled library and its associated packing plasmids pCMV-dR8.2 dvpr and pCMV-VSV-G were obtained from Addgene, lentiviruses were produced by transfecting with HEK293T cells using X-tremeGENE 9 DNA Transfection Reagent from Sigma-Aldrich, and virus was collected 48 h after transfection. Virus was concentrated with Lenti-X Concentrator from Takara and re-suspended in NeuroCult NS-A Complete Medium.

#### CellTag barcode lentivirus transduction and long-term ispinesib treatment

TS543 cells were seeded at ~1000 cells and were transduced with Celltag^26^ virus-laden medium at MOI of around 8–10 and 5 μg/ml protamine sulfate or normal medium and 5 μg/ml protamine sulfate. TS543 were propagated for three weeks before start of treatment. scRNA-seq was performed on drug-naïve TS543. TS543 were treated with 75 nM ispinesib or with vehicle DMSO; medium with ispinesib or DMSO were replenished every two or three days. Viability was monitored as guidepost for resistance. Dead cells were removed with Dead Cell Removal Kit from Miltenyi Biotec; scRNA-seq was initially performed at three-day interval, and later at two- and four-week intervals during treatment.

#### Murine glioma models

For orthotopic cell transplantation experiments, six-week-old CrTac:NCr-Foxn1^nu^ female mice were injected with 5 × 10^4^ cells of last *in vitro* time point of TS543 treated with ispinesib or vehicle DMSO (day 125 control1 samples); ten mice were used per cohort. Mice were clinically monitored daily and sacrificed once end-stage criteria were met, including severe weight loss, seizures, and evidence of motor deficit. Tissues of one mouse from each cohort were harvested and processed for scRNA-seq.

#### Human glioma patient *ex vivo* tissue slices acute drug treatment

Slices were prepared according to the methods outlined in the ‘human glioma patient ex vivo tissue slices’ subsection of the ‘experimental model and study participant details’ section. Acute drug treatment on human glioma patient *ex vivo* tissues slices was performed asfollows.^40^ After the slices were rested for 6 h with the maintenance medium in a humidified incubator at 37°C and 5% CO_2_, the medium was replaced with pre-warmed medium containing drugs with 1.8 nM ispinesib or corresponding volume of vehicle (DMSO). Drug dose was chosen as the estimated IC20 as measured in TS543 patient-derived glioma neurospheres. Slices were then cultured with the treatment medium in a humidified incubator at 37°C and 5% CO_2_ for 18 h before being collected for dissociation.

#### Dissociation of tissue and slices

Mouse brain tumor resections or human glioma patient *ex vivo* tissue slices were dissociated using the Adult Brain Dissociation kit (Miltenyi Biotec) on gentleMACS Octo Dissociator with Heaters (Miltenyi Biotec) according to the manufacturer’s instructions.

#### Microwell scRNA-seq

Microwell 3′ scRNA-seq was performed as follows.^65^ Individual cells were co-encapsulated with a barcoded mRNA capture bead^53^ (MACOSKO-2011-10, ChemGenes) and lysed in microwell-based platform, mRNA transcripts were captured and reverse transcribed on the bead, cDNA-coated beads were pooled for PCR amplification, and Illumina Nextera libraries were constructed for each sample. Gene expression libraries were sequenced on an Illumina NovaSeq 6000 with 51 cycles or 26 cycles for read 1 and 151 cycles for read 2. To sequence the CellTag library separately from the gene expression library at greater sequencing depth, CellTag libraries were constructed with custom P5-TSO_hybrid primer^53^ and custom P7_TruSeq-6bp-Unique-Index_EGFP primer (CAAGCAGAAGACGGCATACGAGAT[6bp-RPI] GTGACTGGAGTTCCTTGGCACCCGAGAATTCCAGGCATGGACGAGCTGTACAAGT*A*A) from the barcoded cDNA libraries. CellTag libraries were sequenced on NextSeq 550 (Illumina) with 26 cycles for read 1 and 58 cycles for read 2.

#### Whole exome sequencing (WES)

Genomic DNA was extracted from cell pellets using the DNeasy Kits (Qiagen) according to the manufacturer’s instructions and was submitted to the Beijing Genomics Institute (BGI) for whole exome sequencing using their DNBseq technology.

#### Dose response assays

TS543 cells were seeded in 96 wells plate at concentration of 1 × 10^4^ cells/cm^2^ and grown for four days and treated with inhibitor for three days. Cell viability was assessed with PrestoBlue Cell Viability Reagent from ThermoFisher to determine the IC50 for each inhibitor. With IC50 as the mid-range concentration, two-drug eight-by-eight dose response checkerboard assays were performed with ispinesib and a candidate inhibitor. TS543 cells were seeded in 384 wells plate at concentration of 1 × 10^4^ cells/cm^2^, grown for four days and treated with ispinesib and candidate inhibitor for three days to assess the drug combination interaction. Ispinesib, URMC-099, WNK463, tomocamycin, NVP231, LMK-235, pentostatin, monensin sodium salt and temozolomide were purchased from MedChemExpress. VBY-825 and V-11-0711 were purchased from AdooQ Bioscience and MedKoo Bioscience respectively.

### QUANTIFICATION AND STATISTICAL ANALYSIS

#### scRNA-seq preprocessing and quality control

Raw data obtained from NovaSeq was corrected for index swapping according to the BarcodeSwapping method.^55^ Raw reads were preprocessed as follows.^5^ Raw reads were subjected to polyA trimming and aligned with STAR. An address comprised of cell-barcode, UMI, and gene identifier was constructed for each read with a unique, strand-specific alignment to exonic sequence. Reads with same address were collapsed and sequencing errors in cell-barcodes and UMI were corrected. Cell-barcodes of empty microwell or low quality cells were removed. Empty cell-barcodes were identified with EmptyDrops algorithm.^56^ Cells were filtered as low-quality if they meet any of the following criteria: 1) fractional alignment to the mitochondrial genome per cell-barcode is greater than 10%, 2) the ratio of molecules aligning to whole gene bodies (including introns) to molecules aligning exclusively to exons is greater than 1.96 standard deviations above the mean, 3) average number of reads per molecule or average number of molecules per gene is greater than 2.5 standard deviations above the mean, or 4) more than 40% of UMI bases are T or where the average number of T-bases per UMI is at least 4.

#### CellTag processing and clone calling

CellTag binary count matrices were generated and clone callings were performed with the CellTagWorkflow algorithm (https://github.com/morris-lab/BiddyetalWorkflow), with an additional preprocessing step of removing UMI with less than three raw reads.

#### WES data processing and Mutect2 analysis

Raw sequencing data were aligned to the human genome (GRCh38) using bwa mem for both the ispinesib-resistant and vehicle-treated samples. Mutations enriched in ispinesib-resistant cells were identified by comparing the two samples using the Genome Analysis Toolkit (GATK).^66^ After duplicate read removal with the Picard *gatk MarkDuplicates* command and quality score adjustment with *gatk ApplyBQSR*, we performed mutation calling with Mutect2 using the *gatk Mutect2* command and Gnomad germline resource af-only-gnomad.vcf.gz and the 1000-genomes panel-of-normals 1000g_pon.hg38.vcf.gz provided by GATK.^67^ In this analysis, the vehicle-treated sample was considered as the “normal” sample and the ispinesib-resistant sample was considered as the “tumor”. After filtering with *gatk GetPipeupSummaries* and *gatk CalculateContamination*, we finalized the variant calls with *gatk FilterMutectCalls*.

#### Identification of malignant glioma cells and non-tumor cells of human glioma *ex vivo* tissue slices

Malignant glioma cells and non-tumor cells of human glioma *ex vivo* tissue slices were identified as follows.^40^ We identified the transformed cells and untransformed cells using the normalized average chromosome 7 and 10 expression in each cell. We first merged scRNA-seq data of all samples derived from the same patient for unsupervised clustering analysis and defined putative malignant cells and non-tumor cells using the genes most specific to each cluster. Putative tumor-myeloid doublet clusters were removed prior to aneuploidy analysis. Next, we computed the average gene expression on each somatic chromosome as follows.^5^ We define the malignancy score to be the log-ratio of the average expression of Chr. 7 genes to that of Chr. 10 genes. We fit a double Gaussian to the malignancy score distribution and established a threshold at 1.96 standard deviations below the mean of the Gaussian with the higher mean (i.e., 95% confidence interval). Putative malignant cells with malignancy scores below this threshold and putative non-tumor cells with malignancy scores above this threshold were discarded as non-malignant or potential multiplets.

#### Differential expression and GSEA

Count matrices for the two conditions were subsampled to give the same cell numbers and same average number of unique transcripts per cell. The resulting count matrix was normalized by the scran deconvolution approach.^68^ Differential expression analysis between two conditions was performed using the Mann-Whitney U-test (*scipy.mannwhitneyu*). The *p*-values were adjusted for false discovery with Benjamini-Hochberg procedure (*statsmodels.multipletests*). Genes were ranked by log_2_(fold change) × −log_10_(FDR adjusted *p*-value). Preranked GSEA was performed on the ranked genes from differential expression analysis with gene sets created from the top scoring genes of scHPF factors. The Normalized Enrichment Score (NES) of GSEA quantifies the representation of gene sets, with positive NES indicating enrichment and negative NES indicating depletion; significance is reported as FDR q-values.

#### Single-cell Hierarchical Poisson Factorization (scHPF) and factor profiling in the TS543 long-term time course

Utilizing single-cell RNA-seq data spanning all four replicates (control1, control2, celltag1, celltag2) across the entire time-course study, we applied single-cell hierarchical Poisson factorization (scHPF)^27^ to construct a comprehensive factor model. The scHPF algorithm yielded a catalog of factors along with their associated gene scores for individual genes (as presented in Figure S1A and Table S1) and cell scores for each cell per factor, where a higher score denotes a greater association with the respective factor. In the subsequent analysis, we excluded factors predominated by ribosomal genes, indicative of sequencing coverage artifacts, and those enriched in interferon-stimulated genes, likely reflecting lentiviral transduction effects. For factor annotation, we sought factors that exhibited high gene scores in correspondence with the Neftel glioblastoma-associated gene sets,^2^ depicted in Figure S1B, to ensure relevance to the glioblastoma phenotype.

#### Single-cell Hierarchical Poisson Factorization (scHPF) and factor characterization of myeloid cells in TS543 neurosphere-derived xenografts

We used the scRNA-seq data from non-tumoral murine cells in TS543 ispinesib-resistant and DMSO-natural-drift neurosphere-derived xenografts to construct a factor model using scHPF.^27^ Myeloid cell populations were identified by clustering with phenograph and visualized with UMAP, guided by scHPF factor scores corresponding to myeloid-specific genes (Cd14, Aif1, Tyrobp, C1qa). Factors derived from the non-tumor scHPF, showing heightened scores specifically within these myeloid clusters, were designated as myeloid-associated factors. We distinguished factors uniquely enriched in the ispinesib-resistant context from those in the DMSO-natural-drift setting by comparing cell scores of factors and by performing differential expression analysis between the myeloid cells of the two xenograft conditions. GSEA was conducted using MSigDB, along with Bowman et al. gene sets derived from bone marrow macrophages and tissue-resident microglia,^39^ to elucidate distinctive gene expression patterns of the two enriched factors.

#### Comparative analysis of acute ispinesib treatment in patient glioma slice cultures and TS543 long-term time-course

To assess the parallelism between glioma patient-derived *ex vivo* tissue slice cultures and TS543 *in vitro* time course models in response to ispinesib treatment, transformed GBM cells were extracted from the patient slice culture data and were projected into the scHPF model based on the TS543 long-term time course. Factors from the scHPF model that were not expressed in the slice culture data (median scHPF cell score <0.02) were removed from subsequent analysis. To measure the similarity in drug response, we calculated the mean fold change in cell scores of ispinesib treatment relative to vehicle, for both tissue slices and TS543 samples, by averaging the ratio of ispinesib-treated to vehicle-treated cell scores. Spearman’s correlation was employed to compare mean cell score fold changes across the datasets. Differential expression analysis between ispinesib-treated and vehicle-treated slice cultures was conducted, followed by GSEA to determine the enrichment scores of the unified MES/AC factor and the Cell Cycle factor within the differentially expressed gene. The unified MES/AC factor was formulated by amalgamating MES/AC-associated factors, from the TS543 *in vitro* time course scHPF model, displaying projection onto the slice culture data.

#### Murine glioma models survival analysis

Survival curves were modeled by Kaplain-Meier method *(lifelines.KaplanMeierFitter)*, and *p*-values were computed by log-rank test *(lifelines.statistics.pairwise_logrank_test).*

#### Drug synergy analysis

Single drug dose-response curves of single drug response assays were fitted with the Hill model (*synergy.single.Hill*) to determine the IC50 of each iinhibitor. Two-drug dose response surfaces of two-drug eight-by-eight dose response checkerboard assays were fit with the MuSyc model^43^ (*synergy.combination.MySyc*), and drug interaction parameters were determined for each drug combination. Parameters are presented as median values with 95% bootstrap confidence intervals, based on bootstrap resampling.

### ADDITIONAL RESOURCES

This paper contains no additional resources.

## Supplementary Material

1

2

3

## Figures and Tables

**KEY RESOURCES TABLE:** 

REAGENT or RESOURCE	SOURCE	IDENTIFIER

Biological samples		

Human glioma patient surgical tumor specimens	Columbia University Irving Medical Center	CUMC5884, CUMC5886, CUMC5944, CUMC5966, CUMC5979

Chemicals, peptides, and recombinant proteins		

ispinesib	MedChemExpress	HY-50759
URMC-099	MedChemExpress	HY-12599
WNK463	MedChemExpress	HY-100626
tomocamycin	MedChemExpress	HY-103248
NVP231	MedChemExpress	HY-13945
LMK-235	MedChemExpress	HY-18998
pentostatin	MedChemExpress	HY-A0006
monensin sodium salt	MedChemExpress	HY-N0150
VBY-825	AdooQ Bioscience	A21069
V-11-0711	MedKoo Bioscience	407806
temozolomide	MedChemExpress	HY-17364

Critical commercial assays		

NeuroCult^™^ NS-A Proliferation Kit Human	STEMCELL Technologies	05751
X-tremeGENE^™^ 9 DNA Transfection Reagent	Sigma-Aldrich	6365779001
Lenti-X^™^ Concentrator	Takara	631231
Adult Brain Dissociation kit	Miltenyi Biotec	130-107-677
Dead Cell Removal Kit	Miltenyi Biotec	130-090-101
PrestoBlueTM Cell Viability Reagent	Invitrogen	P50200
barcoded mRNA capture bead	ChemGenes	MACOSKO-2011-10
Nextera XT DNA Library Preparation Kit	Illumina	FC-131-1024

Deposited data		

Raw and processed scRNAseq data of 1) long-term time course of TS543 2) TS543-derived xenograft 3) ispinesib-treated glioma patient *ex vivo* tissue slices (CUMC5886, CUMC5944, CUMC5966, and CUMC5979)	This paper	GEO: GSE239651
Raw and processed scRNAseq data of vehicle-treated glioma patient *ex vivo* tissue slices 1) vehicle-treated glioma patient *ex vivo* tissue slices (CUMC5884, CUMC5886, CUMC5944, CUMC5966, and CUMC5979) 2) ispinesib-treated glioma patient *ex vivo* tissue slices (CUMC5884)	Zhao et al.^40^	GEO: GSE148842

Experimental models: Cell lines		

Human: TS543	Silber et al.^25^	N/A
Human: HEK293T	Millipore Sigma	12022001-1VL

Experimental models: Organisms/strains		

Mouse: CrTac:NCr-Foxn1^nu^ Female	Taconic Biosciences	NCRNU-F

Oligonucleotides		

P7_TruSeq-6bp-Unique-Index_EGFP primer (CAAGCAGAAGACGGCATACGAGAT[6bp-RPI] GTGACTGGAGTTCCTTGGCACCCGAGAATTCCAGGCATGGACGAGCTGTACAAGT*A*A)	This paper	N/A
P5-TSO_Hybrid primer (AATGATACGGCGACCACCGAGATCTACACGCCTGTCCGCGGAAGCAGTGGTATCAACGCAGAGT*A*C)	Macosko et al.^53^	N/A

Recombinant DNA		

pSMAL-CellTag-V1	Biddy et al.^26^	Addgene_115643
pCMV-dR8.2 dvpr	Stewart et al.^54^	Addgene_8455
pCMV-VSV-G	Stewart et al.^54^	Addgene_8454

Software and algorithms		

DropSeqPipeline8	Yuan et al.^5^	https://github.com/simslab/DropSeqPipeline8
BarcodeSwapping	Griffiths et al.^55^	https://github.com/MarioniLab/BarcodeSwapping2017
EmptyDrops	Lun et al.^56^	https://github.com/MarioniLab/DropletUtils/tree/devel
CellTagWorkflow	Biddy et al.^26^	https://github.com/morris-lab/BiddyetalWorkflow
Single-cell Hierarchical Poisson Factorization	Levitin et al.^27^	https://github.com/simslab/scHPF
Synergy	Wooten et al.^43^	https://github.com/djwooten/synergy
Lifelines	Davidson-Pilon^57^	https://github.com/CamDavidsonPilon/lifelines
Scipy	Virtanen et al.^58^	https://github.com/scipy/scipy
Statsmodels	Seabold et al.^59^	https://github.com/statsmodels/statsmodels
GSEA	Subramanian et al.^60^ and Mootha et al.^61^	https://www.gsea-msigdb.org/gsea/index.jsp
UMAP	McInnes et al.^62^	https://github.com/lmcinnes/umap/tree/master
Phenograph	Levine et al.^63^	https://github.com/jacoblevine/PhenoGraph

## Highlights

- Ispinesib halts mesenchymal shift and favors proneural traits in GBM cells *in vitro*
- Ispinesib-resistant cells form less aggressive xenografts with enhanced survival
- Drug-naive GBM clones exhibit diverse phenotypes, forecasting ispinesib resistance
- Lineage tracing identifies targets for combination therapy with ispinesib
