# Supplementary material for: Multiplexed single-cell lineage tracing of mitotic kinesin inhibitor resistance in glioblastoma
Source: Cell Rep. Author manuscript; Available in PMC 2024 Jun 25. (PMC11199018; doi:10.1016/j.celrep.2024.114139)
Supplement: 1 [file NIHMS2000721-supplement-1.pdf]

Cell Reports, Volume *43*

Supplemental information

Multiplexed single-cell lineage tracing

# of mitotic kinesin inhibitor

resistance in glioblastoma

Yim Ling Cheng, Matei A. Banu, Wenting Zhao, Steven S. Rosenfeld, Peter Canoll, and Peter A. Sims

Supplementary Materials for: Multiplexed single-cell lineage tracing of mitotic kinesin inhibitor resistance in glioblastoma

SUPPLEMENTARY FIGURES

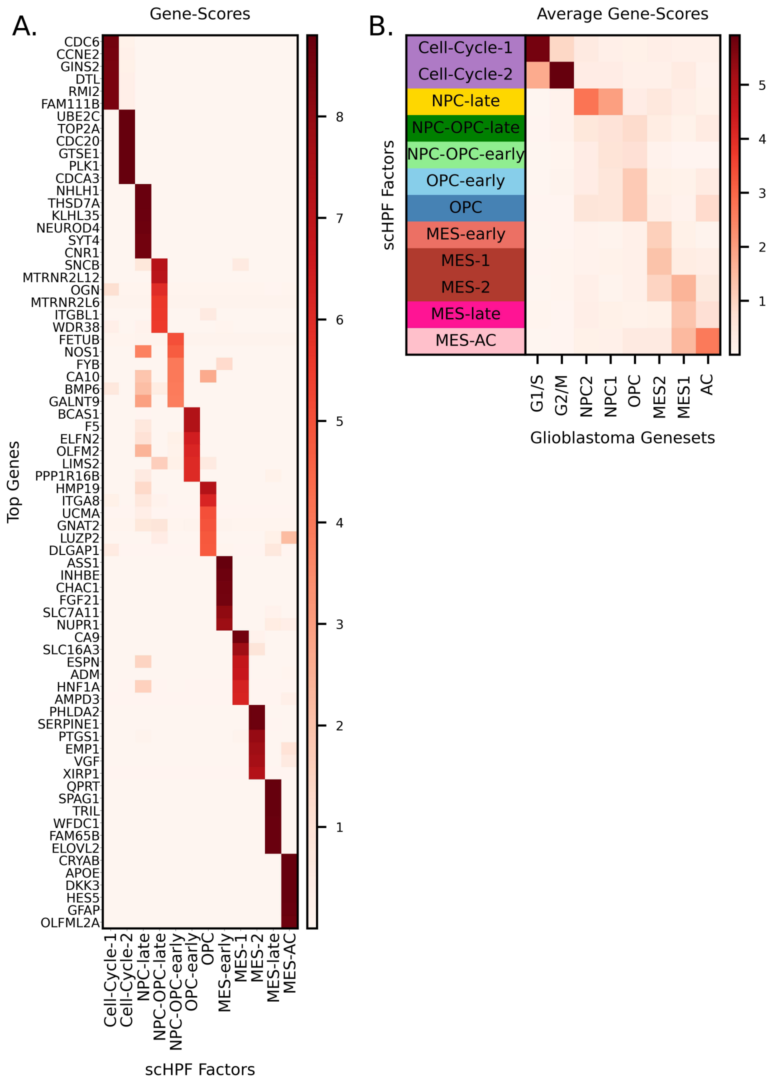

Figure S1. scHPF model of the pooled scRNA-seq data for the *in vitro* time course, related to Figure 1. A) Heatmap of gene-scores of top-ranked genes of scHPF factors. B) Mean scHPF factor gene-scores for Neftel-glioblastoma gene sets.

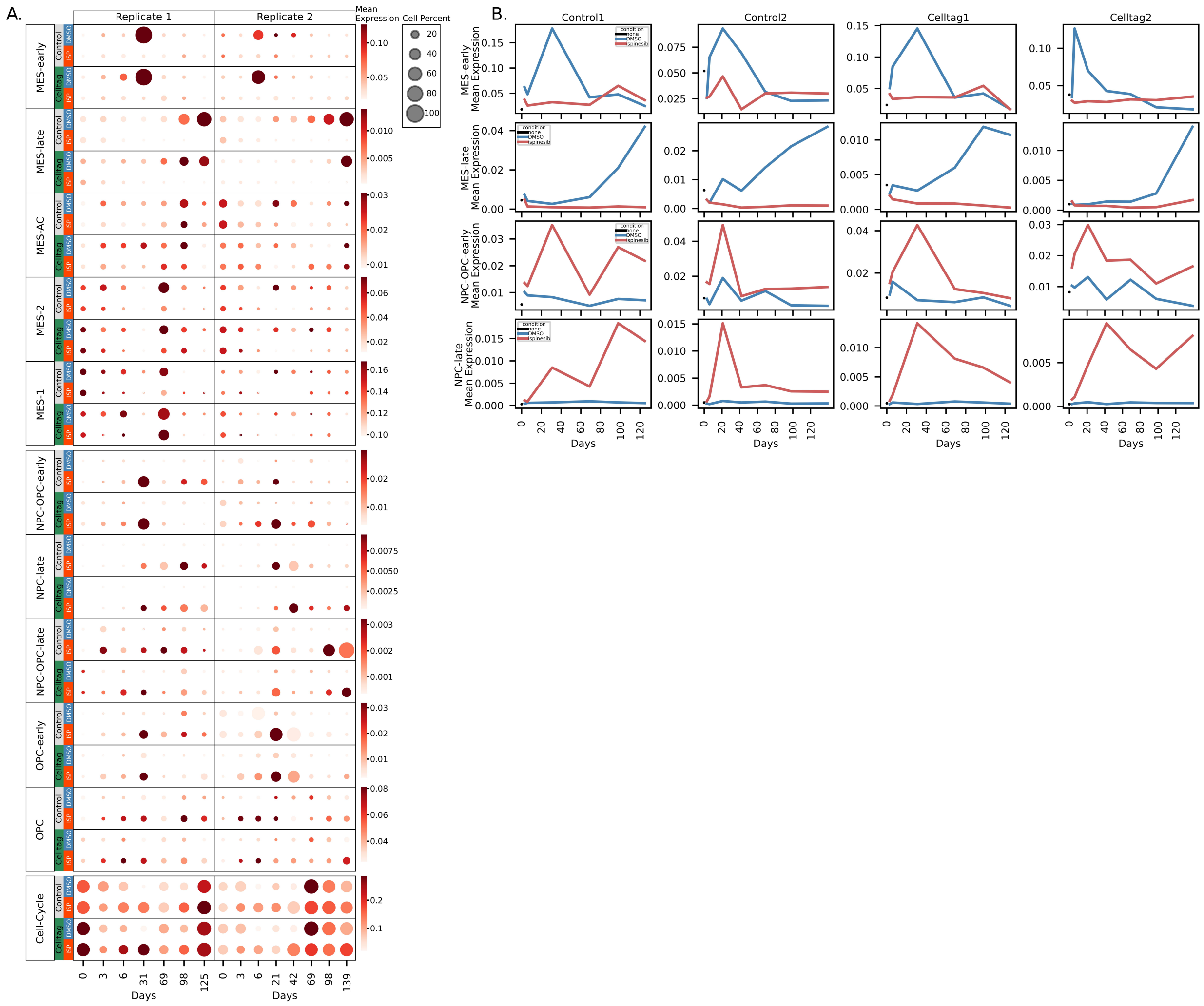

Figure S2. Phenotypic changes for DMSO and ispinesib-treated time course datasets were reproducible across four replicates, related to Figure 1. A) Dot plots of scHPF factors with size indicating the percentage of cells with high cell-scores for the factor and color gradient indicating the mean log-normalized gene expression of top-ranked genes of the factor. B) Line plots of mean log normalized gene expression of top-ranked genes in MES-early factor, MES-late factor, NPC-OPC-early factor, and NPC-late factor.

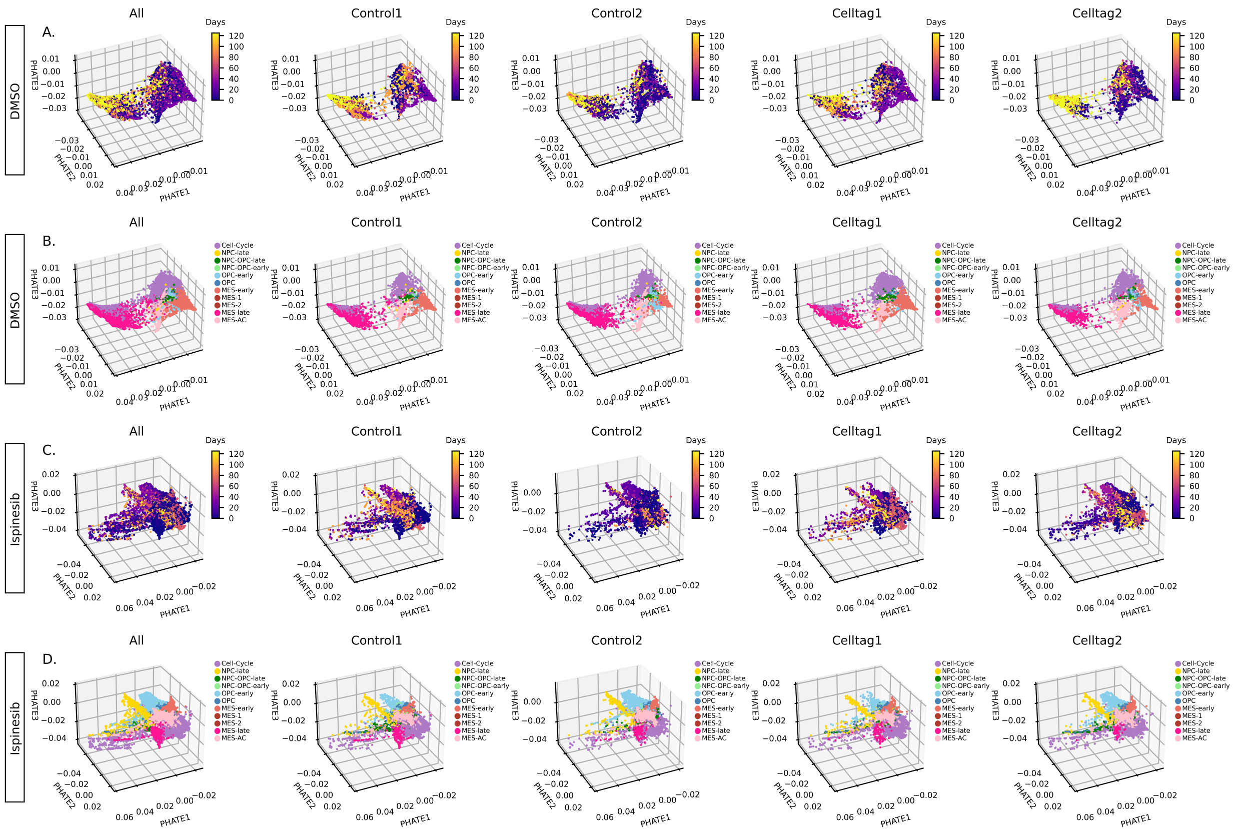

Figure S3. Trajectories of DMSO and ispinesib-treated time course datasets were reproducible across four replicates, related to Figure 1. PHATE embeddings of DMSO samples' scHPF factors (A-B) and ispinesib samples' scHPF factors (C-D), color-coded by treatment days and cell states.

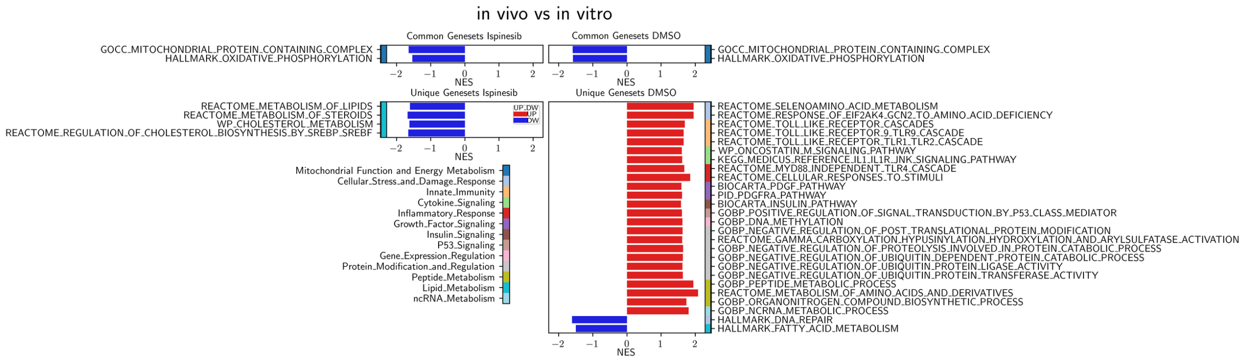

Figure S4. Differential gene set enrichment in ispinesib-resistant and DMSO-natural-draft neurospheres-derived xenografts versus their *in vitro* counterparts, related to Figure 2. GSEA Normalized Enrichment Scores (NES) for MSigDB gene sets significantly affected in (1) *in vivo* DMSO-natural-drift-neurospheres-derived xenograft vs*. in vitro* DMSO-natural-drift neurospherea TS543, and/or (2*) in vivo* ispinesib-resistant-neurospheres-derived xenograft vs. *in vitro* ispinesib-resistant-neurospheres TS543. Analysis highlights three categories: common gene sets significant in both comparisons, unique to ispinesib, and unique to DMSO, with significant NES (q-value < 0.05). Significance is reported as FDR q-values from GSEA.

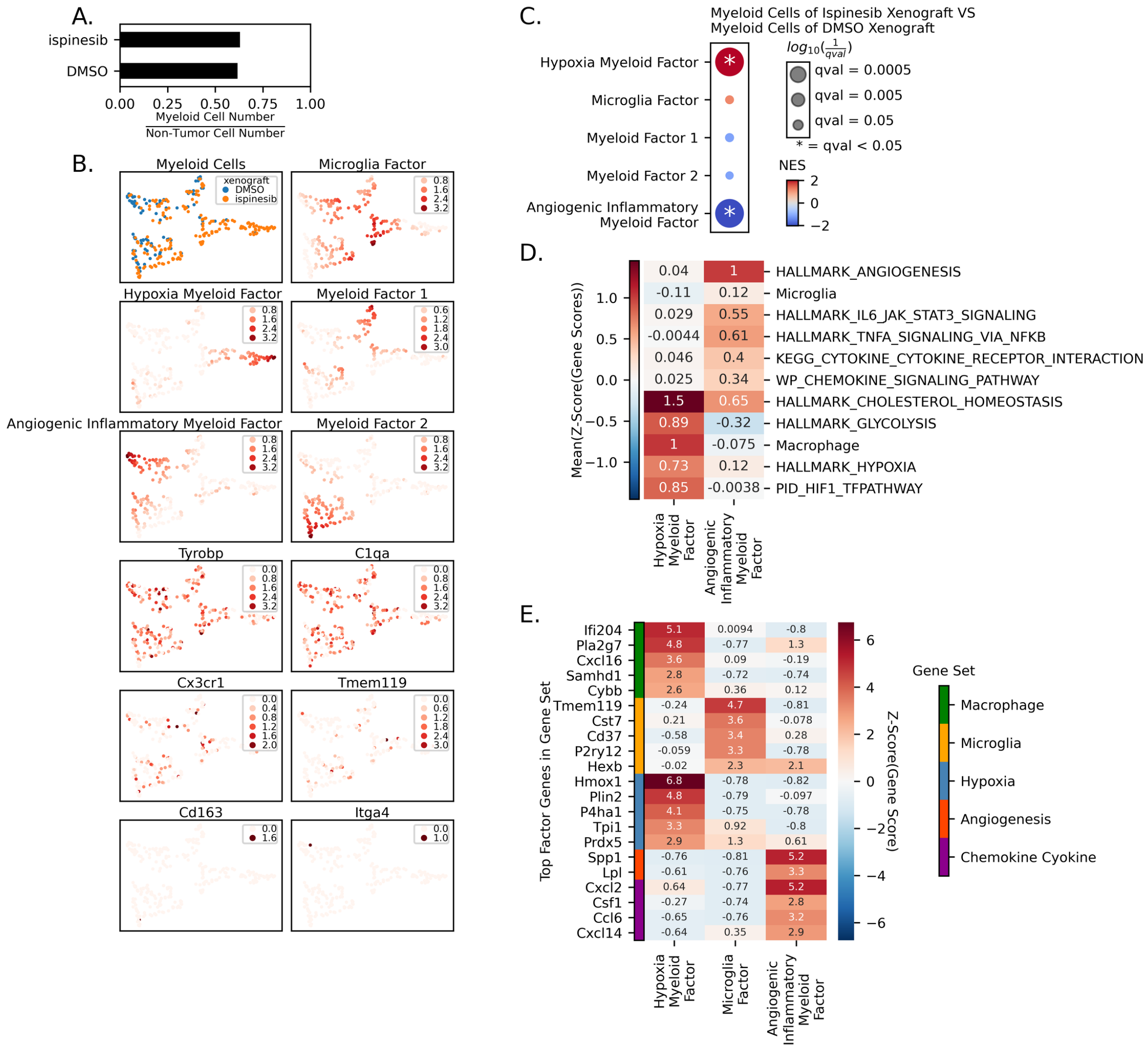

Figure S5. Differential enrichment of hypoxia and angiogenic factors in myeloid cells from ispinesib-resistant and DMSO-natural-drift-derived xenografts respectively, related to Figure 2. A) Myeloid cell proportions in ispinesib-resistant and DMSO-natural-drift neurosphere-derived xenografts' non-tumor (mouse) cells. B) UMAP embeddings displaying myeloid cells from ispinesib-resistant and DMSO-natural-drift xenografts. Cells are color-coded to identify xenograft origins, cell scores for myeloid associated scHPF factor (Hypoxic Myeloid, Angiogenic Inflammatory Myeloid, Microglia, Myeloid Factors 1 and 2), and log-normalized gene expression levels of myeloid (Tyrobp, C1qa), microglia (Cx3cr1, Tmem119), and macrophage markers (Cd163, Itga4). C) GSEA results for differential expression between ispinesib-resistant and DMSO-natural-drift xenografts' myeloid cells, with gene sets derived from top genes of scHPF myeloid factors showing significant enrichment of the Hypoxia Myeloid Factor in the ispinesib-treated sample (red) and depletion of the Angiogenic Inflammatory Myeloid Factor (blue). Significance is reported as FDR q-values from GSEA. D) Z-score transformed scHPF gene scores of myeloid cell factors, highlighting gene sets with differential gene scores for the Hypoxia Myeloid Factor and the Angiogenic Inflammatory Myeloid Factor. E) Z-score transformed gene scores of genes from gene sets of bone marrow macrophages (Macrophage gene set), tissue-resident microglia (Microglia gene set), as well as gene sets associated with

hypoxia, angiogenesis, and chemokine and cytokine signaling listed in D) for Hypoxia Myeloid Factor, Microglia Factor, and Angiogenic Inflammatory Myeloid Factor.

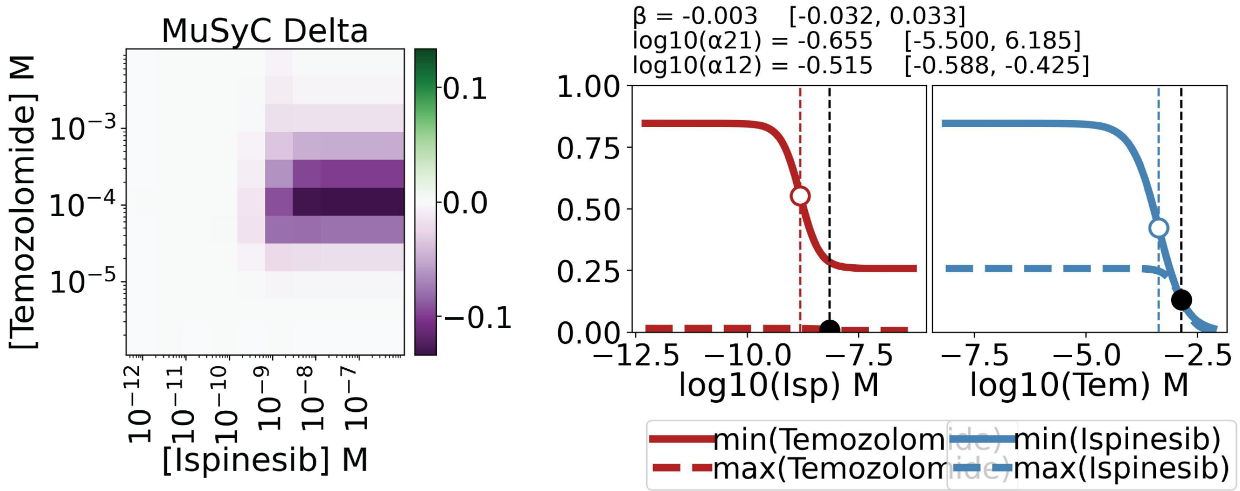

Figure S6. Antagonistic interaction between temozolomide and ispinesib, related to Figure 5. MuSyC Delta heatmap of the difference between the predicted and actual efficacies of ispinesib and temozolomide combination therapy. Side-by-side single drug response curves (solid curve line, with the other drug concentration at zero) and combined drugs response curves (dash curve line, with the other drug concentration at the maximum tested). Parameters are presented as median values with 95% bootstrap confidence intervals, based on bootstrap resampling.

SUPPLEMENTARY TABLES AND LEGENDS

Table S1. Excel sheet containing the top 100 genes in each factor for the scHPF model of the *in vitro* time course, related to Figure 1.

Table S2. Excel sheet containing the top SNVs identified by Mutect2 that are enriched in ispinesib-resistant vs. vehicle-treated TS543 neurospheres based on whole exome sequencing, related to Figure 1.

Sample	Age	Sex	Location	Diagnosis	IDH1 Status	EGFR status	Samples
CUMC5886	50-59	F	splenial glioma extension into left parietal	Glioblastoma, WHO grade IV	wt	amplified	1 vehicle slice, 1 ispinesib slice
CUMC5884	60-69	M	right parietal	Glioblastoma, WHO grade IV	wt	unamplified	2 vehicle slices, 1 ispinesib slice
CUMC5944	60-69	M	left frontal	Glioblastoma, WHO grade IV	wt	amplified	3 vehicle slices, 1 ispinesib slice
CUMC5966	60-69	F	left parieto-occipital	Glioblastoma, WHO grade IV	wt	unamplified	2 vehicle slices, 1 ispinesib slice
CUMC5979	50-59	M	right temporal	Glioblastoma, WHO grade IV	wt	amplified	2 vehicle slices, 1 ispinesib slice

Table S3. Summary of patients and specimens used for scRNA-seq of *ex vivo* slice cultures, related to Figure 3.
